# Severe Delirium and Refractory Bradycardia Associated With Uncaria tomentosa Toxicity: A Case Report

**DOI:** 10.7759/cureus.114193

**Published:** 2026-08-09

**Authors:** Richard Giovane

**Affiliations:** 1 Family Medicine, The University of Alabama, Tuscaloosa, USA

**Keywords:** dietary supplement, drug-induced bradycardia, drug-induced psychosis, first-degree heart block, sick sinus syndrome

## Abstract

*Uncaria tomentosa* (cat’s claw) is an Amazonian medicinal vine widely used for its anti-inflammatory properties. While generally safe at therapeutic doses, its active oxindole alkaloids can precipitate profound cardiovascular and central nervous system (CNS) toxicity in supratherapeutic amounts. We present a case of a 53-year-old female who presented with severe insomnia, anxiety, and gastrointestinal distress following an attempt to self-wean from a high-dose, over-the-counter cat’s claw supplement. Her hospital course was complicated by severe delirium requiring intensive care and persistent, symptomatic sinus bradycardia, 29-40 beats per minute with first-degree atrioventricular (AV) block. Although her neuropsychiatric symptoms resolved with supportive care and benzodiazepines, her bradycardia remained refractory to medical management. Subsequent electrophysiology evaluation was suggestive of sick sinus syndrome, which was successfully treated with the implantation of a permanent dual-chamber pacemaker. This case highlights the potential for unregulated herbal supplements to induce severe neuro-cardiac toxicity and unmask underlying cardiopulmonary disease. Clinicians must maintain a high index of suspicion for toxicological etiologies and underlying structural pathologies when evaluating complex, multisystem presentations.

## Introduction

*Uncaria tomentosa*, commonly known as cat’s claw, is a medicinal climbing vine indigenous to the Amazon rainforest, predominantly utilized for its anti-inflammatory and immunomodulatory properties in managing musculoskeletal conditions [1]. While generally tolerated at standard therapeutic doses, its pharmacological activity is driven by a dense profile of oxindole alkaloids. Unregulated formulations can lead to supratherapeutic ingestion of these compounds, precipitating severe cardiovascular and central nervous system (CNS) toxidromes. We present a complex case of a patient exhibiting severe delirium and symptomatic bradycardia following the ingestion of a cat's claw-containing supplement, which was suggestive of unmasking an underlying sick sinus syndrome requiring permanent pacing.

## Case presentation

A 53-year-old female with a history of left-sided carpal tunnel syndrome presented to the emergency department reporting four days of absolute insomnia, severe anxiety, racing thoughts, anorexia, and gastrointestinal distress. She noted that any attempt to rest triggered worsening anxiety and racing thoughts.

The patient reported a one-month history of taking an over-the-counter supplement named “Visors,” obtained from a gas station for pain control. The product packaging identified cat’s claw as the primary ingredient in a proprietary blend, with other ingredients being preservatives. She initially consumed 10-12 tablets daily (packaged as four tablets per blister pack) but had been self-weaning over the preceding weeks, which precipitated her escalating neuropsychiatric symptoms. It should be noted that the patient was unsure of the self-weaning dose each day; however, reported that she tried to take fewer tablets each day. Of note, the patient mentioned that a store attendant suggested the product might contain synthetic opioids. The patient did bring a package of the supplement, which had no manufacturer's labelling. Due to her worsening symptoms, she came to the emergency room for further evaluation. On presentation, the patient's vital signs were stable: blood pressure of 120/77, heart rate of 64 beats per minute (BPM), respiratory rate of 16, and 98.7°Fahrenheit (F).

Regarding social history, the patient denied smoking tobacco, drinking alcohol, or any illicit drug use. Regarding her psychiatric history, the patient reported a remote history of general anxiety disorder, for which she was previously on escitalopram 10 mg; however, she had stopped taking it several months ago.

**Figure 1:**
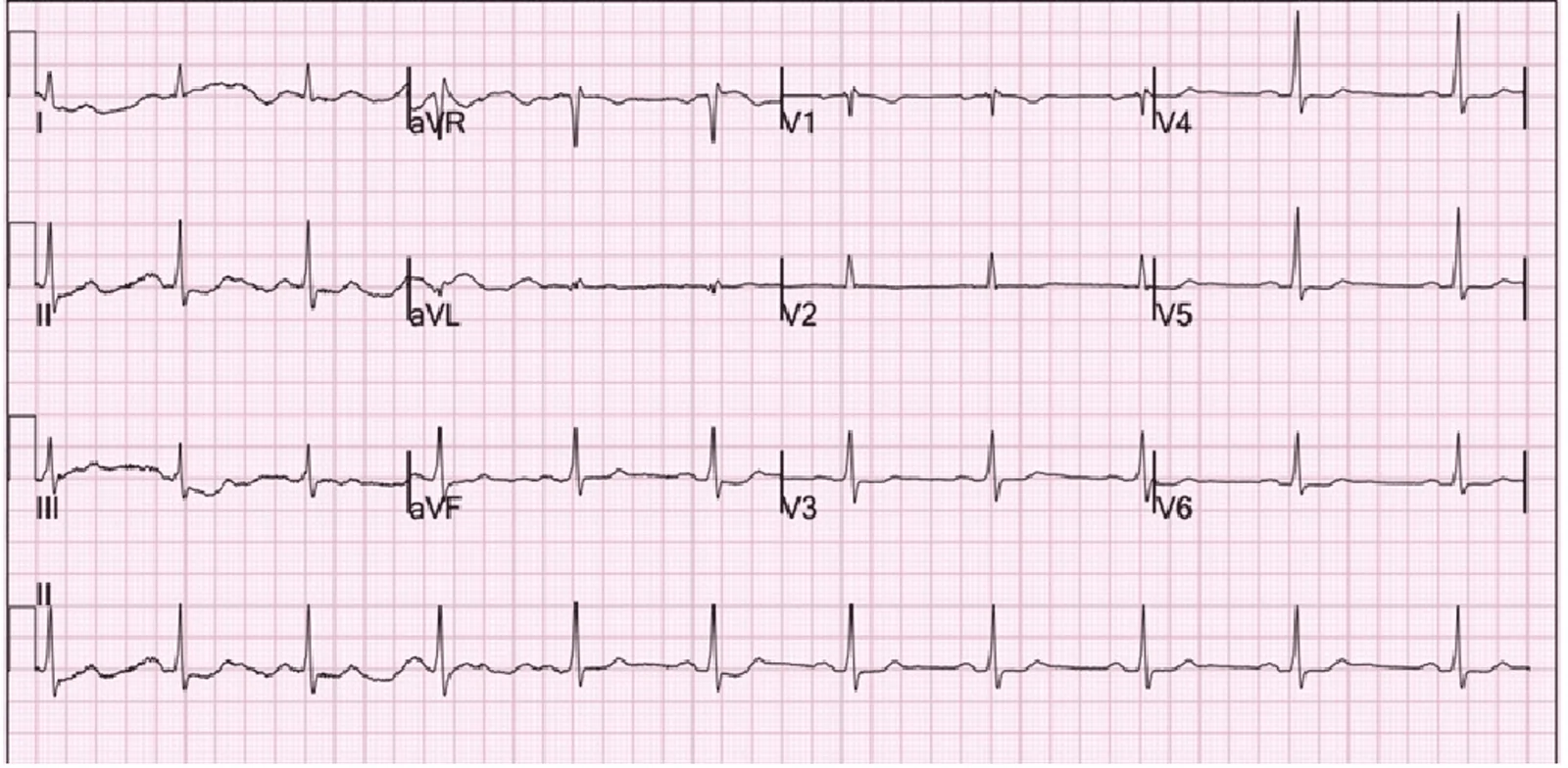
Initial ECG obtained in the emergency room demonstrating sinus rhythm with a heart rate of 63 beats/min, PR interval of 153 ms, QRS duration of 110 ms, and QTc of 433 ms.

Initial complete blood count (CBC) and comprehensive metabolic panel (CMP) were unremarkable. A baseline electrocardiogram (EKG) showed normal sinus rhythm with a normal PR interval (Figure 1).

Poison control was consulted and recommended supportive care with either admission for observation or outpatient management via a chlordiazepoxide taper. Due to the severity of her insomnia and symptom burden, the patient was admitted for observation.

**Figure 2:**
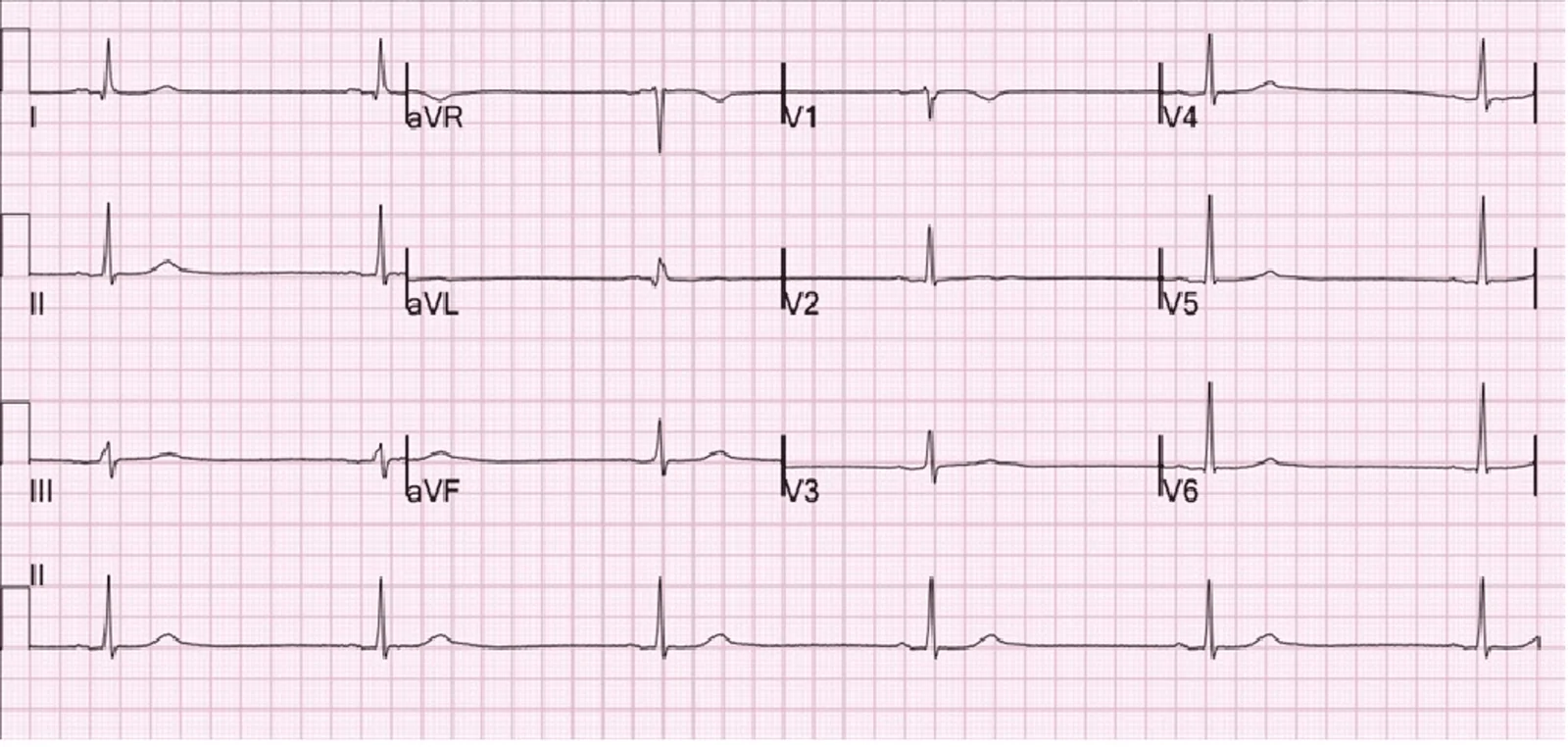
ECG demonstrating sinus bradycardia with first-degree atrioventricular (AV) block and a prolonged corrected QT interval (QTc), with a heart rate of 32 beats/min, PR interval of 205 ms, QRS duration of 102 ms, and QTc of 501 ms.

Before transferring to the medical floor, the patient developed acute agitation and delirium due to the patient being previously alert and oriented to person, place, and time; however, she was then only alert and oriented to person, prompting an escalation of care to the intensive care unit (ICU). She was initially given lorazepam (1 mg hourly, as needed) for agitation. A dexmedetomidine infusion was considered for worsening symptoms; however, prior to its initiation, she exhibited severe sinus bradycardia (29-40 bpm) with a first-degree atrioventricular (AV) block (Figure 2).

Consequently, the patient was started on a midazolam infusion for sedation. Repeat laboratory investigations drawn six hours post-admission revealed mild metabolic derangements, including a bicarbonate level of 18 mEq/L (reference range: 22-29 mEq/L), aspartate aminotransferase (AST) of 70 U/L (reference range: 10-40 U/L), alanine aminotransferase (ALT) of 50 U/L (reference range: 7-56 U/L), calcium of 8.2 mg/dL (reference range: 8.5-10.5 mg/dL), magnesium of 1.8 mg/dL (reference range: 1.6-2.4 mg/dL), thyroid-stimulating hormone (TSH) of 6.08 μIU/mL (reference range: 0.2-4.2 μIU/mL), total thyroxine (T4) of 7.6 μg/dL (reference range: 4.5-12.0 μg/dL), and a white blood cell (WBC) count of 9.71 × 10^3^/μL (reference range: 4.0-10.0 × 10^3^/μL).

**Figure 3:**
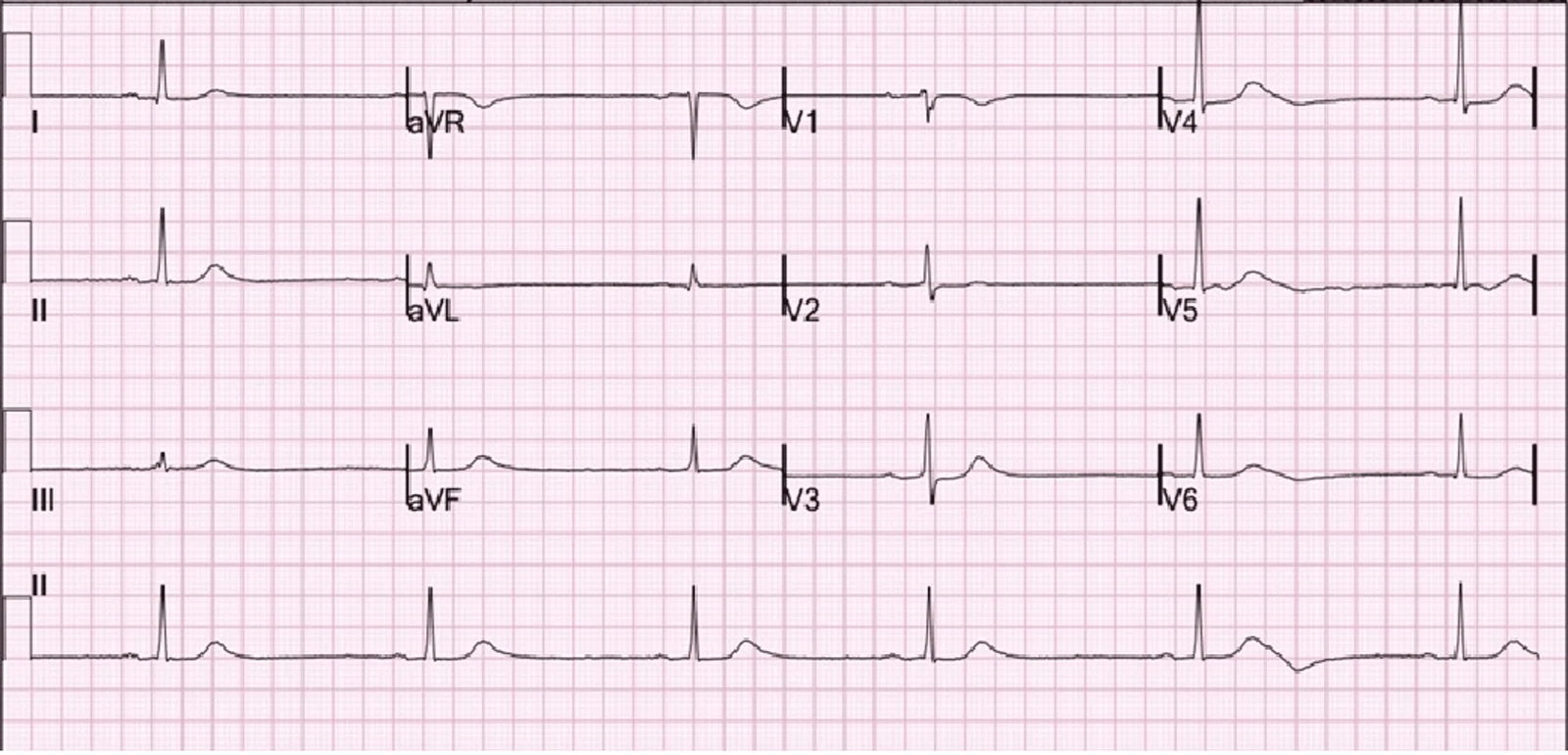
ECG demonstrating persistent sinus bradycardia with first-degree atrioventricular (AV) block and a prolonged corrected QT interval (QTc), with a heart rate of 34 beats/min, PR interval of 220 ms, QRS duration of 96 ms, and QTc of 539 ms.

Cardiac enzymes trended over six hours (three sets) were negative, and chest radiography showed no acute cardiopulmonary process. With supportive care and gradual weaning of midazolam, the patient’s mental status steadily improved over 24 hours. However, she demonstrated persistent, symptomatic sinus bradycardia (heart rates in the 30s-40s) despite the discontinuation of the offending supplement for >72 hours (Figure 3).

Cardiology was consulted and administered a trial dose of atropine. Her heart rate transiently increased to the 70s before dropping back to the 30s-40s. A dopamine infusion was subsequently initiated for chronotropic support, which successfully maintained her heart rate in the 70s-80s; however, attempts to wean the dopamine resulted in recurrent, severe bradycardia.

When more history could be obtained from the patient after her psychosis resolved, further history revealed longstanding, pre-admission symptoms of presyncope/syncope and a significant family history of pacemaker placement. Electrophysiology (EP) was consulted. While initial consideration was given to reversible/toxic causes, the persistence of her bradycardia and her clinical history raised strong suspicion for underlying sinus node dysfunction.

**Figure 4:**
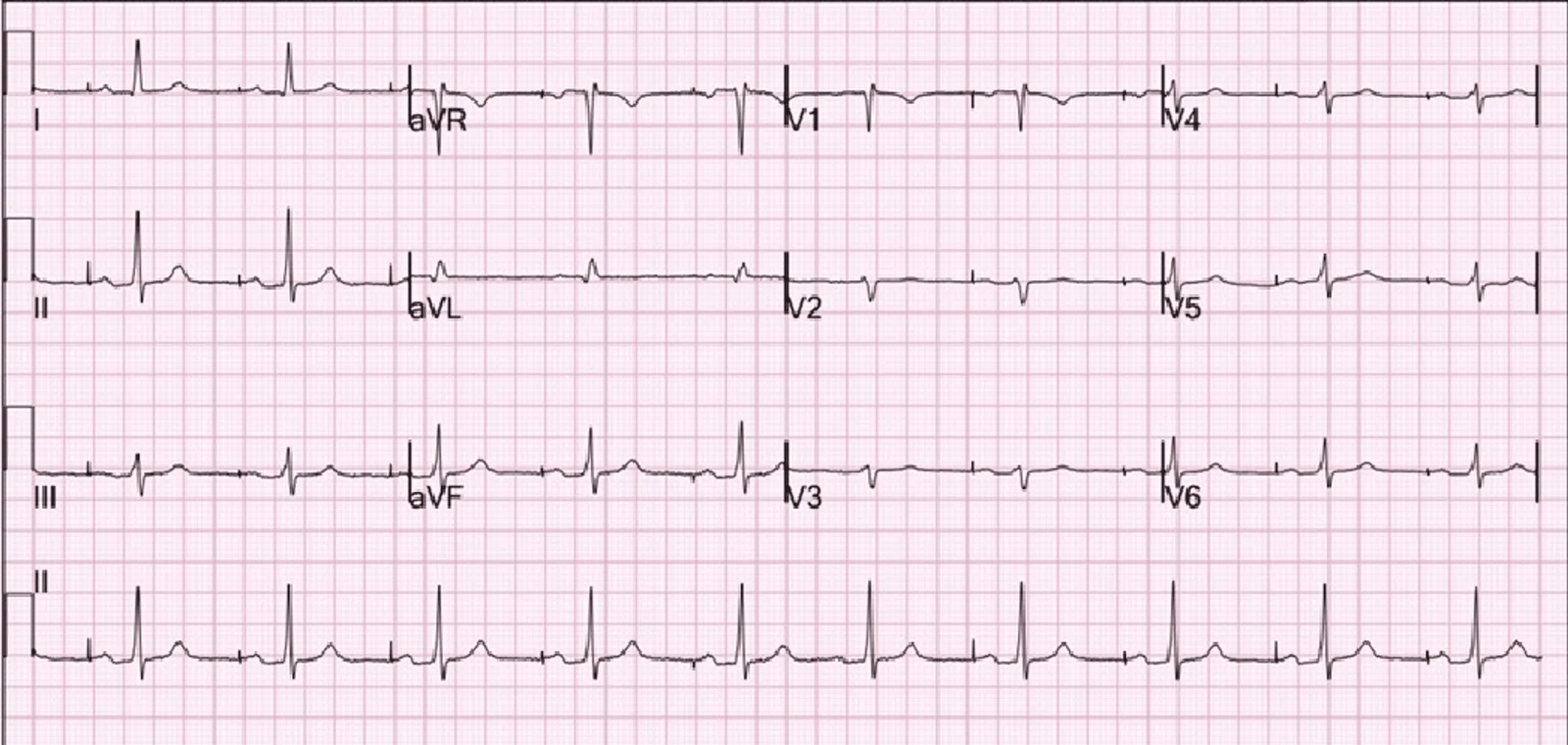
ECG obtained after pacemaker implantation demonstrating an atrial-paced rhythm, with a heart rate of 60 beats/min, PR interval of 192 ms, QRS duration of 100 ms, and corrected QT interval (QTc) of 420 ms.

Given the refractory nature of her symptomatic bradycardia, the patient underwent successful implantation of a Medtronic dual-chamber permanent pacemaker (Medtronic plc, Minneapolis, MN, USA) in the left prepectoral pocket under fluoroscopic guidance. Following implantation, her heart rate stabilized between 60 and 80 bpm (atrially paced) (Figure 4), and her bradycardic symptoms resolved.

Her neuropsychiatric symptoms completely resolved with supportive care by the time the pacemaker was placed. She remained hemodynamically stable on room air. Aside from mild anemia and minor electrolyte variations that did not require intervention, her remaining hospital course was uneventful. She was discharged with close EP follow-up; a two-week follow-up appointment confirmed proper pacemaker function and no recurrent issues.

## Discussion

This case illustrates a dual presentation of first-degree AV block and acute psychosis following a potential toxicity, highlighting the danger of unregulated herbal supplements in unmasking underlying cardiopulmonary disease. The patient's symptoms can be mechanistically linked to the known pharmacodynamics of *U. tomentosa*.

The cardiovascular toxicity of cat's claw in a supratherapeutic setting closely mimics the pharmacology of a non-dihydropyridine calcium channel blocker [1,2]. The plant's bioactivity is driven by pentacyclic oxindole alkaloids (POAs) and tetracyclic oxindole alkaloids (TOAs), specifically rhynchophylline, isorhynchophylline, and hirsutine. Rhynchophylline and isorhynchophylline act as potent L-type calcium channel blockers [2]. By inhibiting the influx of extracellular calcium in cardiac myocytes and nodal tissue, these alkaloids suppress the vasomotor center and induce significant negative chronotropic and dromotropic effects [3,4]. In some studies, in vivo models demonstrate that toxic doses of these specific *Uncaria* alkaloids rapidly induce profound bradycardia and prolonged action potential durations [3]. Hirsutine also acts synergistically on cardiovascular targets to lower systemic blood pressure [4].

In regards to the manifestation of delirium, although rarer, pharmacologically established consequences of oxindole alkaloid toxicity can still occur. Because these alkaloids are highly lipophilic, they readily cross the blood-brain barrier [2]. High systemic concentrations of POAs and TOAs have been shown to interact directly with central dopamine and serotonin (5-HT) receptors [5]. Toxicological studies on *U. tomentosa* extracts demonstrate that extreme doses induce acute behavioral alterations attributed to dopaminergic overstimulation [6]. The oxindole alkaloids in cat's claw share structural and functional similarities with alkaloids found in *Mitragyna speciosa *(Kratom), such as corynoxeine [5,6]. In cases of overdose or abuse, these related alkaloids exhibit known psychotropic properties capable of precipitating altered mental status, delirium, and severe withdrawal-like states upon cessation.

In this patient, the massive ingestion of these alkaloids likely induced toxicity, which in turn likely unmasked underlying sick sinus syndrome. More interestingly, the persistence of the bradycardia beyond the expected clearance window of the supplement served as a critical diagnostic clue, revealing her underlying, previously asymptomatic sick sinus syndrome.

It is highly probable that the patient's acute psychiatric presentation was driven by this central alkaloid surge, compounded by acute cerebral hypoperfusion secondary to her profound bradycardia. While the patient noted the potential presence of synthetic opioids in the supplement, her clinical presentation - specifically the absence of respiratory depression and the presence of refractory bradycardia and agitation - aligns heavily with oxindole alkaloid toxicity.

## Conclusions

This case highlights a multifactorial presentation involving suspected herbal supplement toxicity, withdrawal, severe bradycardia, and delirium. While the initial clinical picture heavily suggested a reversible toxicological etiology, the presence of persistent bradycardia changed this narrative due to the patient still having cardiac symptoms. It prompted further electrophysiological evaluation, ultimately diagnosing and treating sick sinus syndrome. This case challenges clinicians to evaluate the use of over-the-counter supplementation as a means of unmasking an underlying pathology.
